# Characterization of the functions and proteomes associated with membrane rafts in chicken sperm

**DOI:** 10.1371/journal.pone.0186482

**Published:** 2017-11-02

**Authors:** Ai Ushiyama, Atsushi Tajima, Naoto Ishikawa, Atsushi Asano

**Affiliations:** 1 Graduate School of Life and Environmental Sciences, University of Tsukuba, Ibaraki, Japan; 2 Faculty of Life and Environmental Sciences, University of Tsukuba, Ibaraki, Japan; Carl von Ossietzky Universitat Oldenburg, GERMANY

## Abstract

Cellular membranes are heterogeneous, and this has a great impact on cellular function. Despite the central role of membrane functions in multiple cellular processes in sperm, their molecular mechanisms are poorly understood. Membrane rafts are specific membrane domains enriched in cholesterol, ganglioside G_M1_, and functional proteins, and they are involved in the regulation of a variety of cellular functions. Studies of the functional characterization of membrane rafts in mammalian sperm have demonstrated roles in sperm-egg binding and the acrosomal reaction. Recently, our biochemical and cell biological studies showed that membrane rafts are present and might play functional roles in chicken sperm. In this study, we isolated membrane rafts from chicken sperm as a detergent-resistant membranes (DRM) floating on a density gradient in the presence of 1% Triton X-100, and characterized the function and proteomes associated with these domains. Biochemical comparison of the DRM between fresh and cryopreserved sperm demonstrated that cryopreservation induces cholesterol loss specifically from membrane rafts, indicating the functional connection with reduced post-thaw fertility in chicken sperm. Furthermore, using an avidin-biotin system, we found that sperm DRM is highly enriched in a 60 KDa single protein able to bind to the inner perivitelline layer. To identify possible roles of membrane rafts, quantitative proteomics, combined with a stable isotope dimethyl labeling approach, identified 82 proteins exclusively or relatively more associated with membrane rafts. Our results demonstrate the functional distinctions between membrane domains and provide compelling evidence that membrane rafts are involved in various cellular pathways inherent to chicken sperm.

## Introduction

Biological membranes are heterogeneous, and this is critical for cellular function. Membrane rafts are dynamic membrane regions enriched in functional proteins and specific lipids, such as cholesterol and glycosphingolipid G_M1_ (G_M1_). The organization of the membrane domains relies on biochemical interactions among the constituents. Cholesterol is a key molecule regulating both organization and disruption of the micro-domains, suggesting a dynamic appearance in temporal and spatial scales. Attempts to understand the physiological roles of the membrane micro-domains have demonstrated that these domains play significant roles in a variety of cellular functions [1–3]. These tremendous functional aspects of the rafts have made them of great interest for cell biologists. However, there are numerous difficulties associated with observing the nature of raft domains because of their small and dynamic appearance. To avoid these difficulties, biochemical isolation based on resistance to solubilization when incubated at low temperature with Triton X-100 (TX-100) [4] is often preferred as a starting point to explore the presence and compositional nature of membrane rafts. Although this approach led to controversy that these detergent resistant membranes (DRM) might not exactly represent pre-existing rafts in cellular membranes [5], a strong correlation has also been demonstrated between the molecules recovered in the DRM and those partitioned into the raft domains *in situ* [6]. Furthermore, many of major findings for the functional characterization of membrane rafts have originated from DRM-based analysis. Therefore, it has been proposed that DRM-based methodology can be used to identify molecules that tend to be associated with membrane rafts [7].

Of the cell types in which membrane domain organization has been studied, mammalian sperm are unique with respect to the size and stability of the lipid segregations within their plasma membrane [8]. We have previously shown in live murine sperm that an enormous membrane domain is enriched in G_M1_, which is localized to the acrosomal plasma membrane (APM) region [8]. Furthermore, a recent localization experiment demonstrated that the APM region consists of multiple membrane domains with focal enrichments of sterols [9]. Considering that localization is highly conserved between mammalian species [9, 10], these data suggest the presence of membrane rafts in the APM region of mammalian sperm. Several studies have attempted to demonstrate possible roles in mammalian sperm, including capacitation [11, 12], binding to the zona pellucida (ZP)[13, 14], and the acrosome reaction [15, 16], which further makes the functional characterization of sperm rafts of great interest.

Unlike mammalian sperm, there is no capacitation process recognized in avian sperm, but it is in common to undergo the acrosome reaction by binding to the inner perivitelline layer (IPVL), which is considered to be a structure that is analogous to the mammalian ZP [17]. In Japanese quail, it recently was demonstrated that a 45KDa sperm acrosin on APM is responsible for sperm binding to the IPVL of an ovum [18]. Furthermore, the APM contains multiple calcium channels involved in induction of the acrosome reaction [19]. Considering that sperm are transcriptionally and translationally inactive, these highlight the functional role of preassembled cellular machinery into the APM region, as it allows avian sperm to induce fertilization competence. We recently reported in chicken sperm that membrane rafts became localized to the plasma membrane overlying the sperm head, including the APM region [20]. Furthermore, a comparison of membrane properties between fresh and cryopreserved sperm demonstrated that alteration of membrane rafts occurs concomitantly with cholesterol loss during cryopreservation, which results in impairment of post-thaw fertilizing ability [21]. These data suggest that membrane rafts play a more substantial role in regulation of sperm function during fertilization.

Mass spectrometry of the DRM fraction is a powerful tool to screen the functional roles of membrane rafts. Although several studies were performed in sperm DRM to identify potential molecules partitioned into membrane rafts [11, 22, 23], it has been pointed out that the association of molecules with the DRM remains to be determined with quantitative approaches because of uncertainty regarding whether molecules are primarily associated with the DRM or non-DRM [7]. To avoid this limitation, our group has previously performed quantitative proteomic characterization of membrane rafts in murine sperm, highlighting the functional roles of sperm rafts [24]. However, the proteome associated with membrane rafts remain to be characterized in any avian sperm. Therefore, in this study, we isolated the DRM and characterized the possible functions and proteomes of membrane rafts in chicken sperm. Our biochemical and quantitative proteomic data indicate a functional distinction between membrane domains and suggest the functional involvement of membrane rafts in multiple cellular pathways inherent in chicken sperm.

## Materials and methods

### Reagents and animals

All chemicals were purchased from Sigma-Aldrich (St. Louis, MO, USA) unless otherwise noted. The Amplex^Ⓡ^ Red Cholesterol Assay Kit, cholera toxin subunit B conjugated with horseradish peroxidase or AlexaFluor 488 (CTB-HRP or–Alexa 488) and NeutrAvidin^TM^ Agarose were obtained from Thermo Fischer Scientific (Waltham, MA, USA).

Fertile Rhode Island Red chickens, raised at the Agricultural and Forestry Research Center, University of Tsukuba, Japan, were utilized for semen collection using the dorsal-abdominal massage method [25]. In brief, ejaculatory response was induced by the dorsal abdominal massage and then semen was collected by gripping the base of the protruded copulatory organ. All animal work was performed following approval of the University of Tsukuba’s Institutional Animal Care and Use Committee (Approval number 16–011).

### Sperm cryopreservation

Cryopreservation of sperm was performed as described previously [26]. In brief, clean semen samples collected from multiple males were pooled and diluted three times with Minnesota Avian buffer (MnA) containing 8% (v/v) glycerol. Samples were snap frozen in liquid nitrogen for storage after loading into a 0.5 ml straw. For thawing, frozen semen was thawed at 5°C for 10 min. Semen was rediluted, centrifuged, and then resuspended in MnA.

### Separation of the DRM

Membrane rafts were isolated from fresh and thawed sperm as the low density DRM, as described previously [4]. Sperm (1.5×10^8^) were sonicated and treated with ice cold TNE (50 mM Tris, 150 mM NaCl, 1 mM EDTA, pH7.35) containing 1% TX-100 and protease inhibitor cocktail (Roche Applied Science, Indianapolis, IN, USA) for 30 min. After removal of sperm debris by centrifugation at 10,000 g for 10 min, the supernatant was sonicated with three short bursts and then mixed with 60% (w/v) sucrose to obtain a 40% (w/v) final sucrose concentration. This mixture (0.75 ml) was placed at the bottom of the tube and overlaid with 2.4 ml of 30% sucrose and 1.0 ml of 5% sucrose and then centrifuged at 200,000 g for 18 h. Fractions (0.5 ml) were collected from the top (designated 1 to 8 from top to bottom) by careful pipetting and subjected to quantification of cholesterol, G_M1_, and protein amount. The density of each fraction was as follow: fractions 1; 1.016 g/cm^3^, 2; 1.017 g/cm^3^, 3; 1.104 g/cm^3^, 4; 1.131 g/cm^3^, 5; 1.127 g/cm^3^, 6; 1.122 g/cm^3^, 7; 1.142 g/cm^3^, 8; 1.146 g/cm^3^.

### G_M1_ quantification

For the determination of G_M1_ content, fractions were subjected to slot blotting as described [24]. In brief, 25 μl of each fraction was diluted with 75 μl of TNE and was blotted onto a PVDF membrane (Immobilon-P; Millipore, Bedford, MA, USA) using a Slot Blot Manifold (Hoefer, San Francisco, CA, USA). The PVDF membrane was blocked with 5% bovine serum albumin and incubated with CTB-HRP at a 1:2000 dilution for 1 h at room temperature. The G_M1_ expression was detected by chemiluminescence using the ChemiDoc XRS+ (Bio-Rad, Hercules, CA, USA), and the resulting bands were subjected to densitometry using ImageJ 1.47v software downloaded from the NIH website (http://imagej.nih.gov/ij/).

### Immunoblotting

Proteins from the fractions were extracted by boiling in sample buffer [27] and separated by SDS-PAGE. Transfer, blocking, and immunodetection of specific proteins were performed largely as previously described [24]. Dilutions used for the primary antiserum were 1:4,000 for anti-α-tubulin (EMD Millipore, Millerica, MA, USA) and 1:500 for anti-acrosin (Santa Cruz Biotechnology, Dallas, TX, USA), and for the biotin-binding probe was 1:100,000 for NeutrAvidin-HRP (Thermo Fischer Scientific). A 1:5,000 dilution was used for anti-mouse IgG conjugated with HRP (GE Healthcare Life Sciences, Pittsburg, PA, USA). Chemiluminescence was used to detect immunoreactivity.

### Localization of lipids in sperm

Fresh semen and frozen-thawed semen (1 x 10^7^ sperm), which were thawed and suspended in MnA, were centrifugally washed in PBS, and fixed for 15 min at room temperature with 2% paraformaldehyde in PBS. Localization of G_M1_ and sterols was determined as described previously [8]. In brief, the sperm were washed with PBS and incubated with 10 μg/ml CTB-Alexa 488 or 50 μg/ml Filipin III (Cayman Chemical, Ann Arbor, MI, USA) in 300 μl PBS. The sperm were then washed with PBS, and viewed with a Leica DMI 4000 B microscope (Leica Microsystems, Wetzlar, Germany) equipped with Leica DFC 450 camera. Images were captured with the same exposure times for fresh and frozen-thawed sperm.

### Sperm membrane protein-IPVL binding assay

IPVLs were separated from fowl eggs as described [28]. Based on the buoyancy and contents of cholesterol and G_M1_, fraction 2 and 3 represented the putative DRM and were pooled together for a further assay. For assessment of the binding affinity of sperm membrane rafts to the IPVL, proteins of the low density DRM fraction (fractions 2 and 3) and fraction 8 (representing non-rafts) were biotinylated, using EZ-Link^TM^ Sulfo-NHS-LC-Biotinylation Kit (Thermo Fischer Scientific) as described in the manufacture’s instruction. In brief, the samples were subjected to desalting and then to a micro-BCA protein assay. Equivalent amounts of protein were biotinylated and then separated from unbound biotin using a desalting column. A 1 cm^2^ square of IPVL was mounted on a glass slide and co-incubated with 150 μg biotinylated protein overnight at 4°C.

After rinsing with PBS, samples were incubated with FITC-streptavidin (1:70 dilution with PBS) for 1 h at room temperature and a cover slip was mounted using Antifade Mounting Medium (Vector Laboratories, Peterborough, UK). The IPVL was viewed as described above. Images were captured with a constant exposure time. Mean values of fluorescent intensity in the IPVL were computed from the images using Leica AF6000 imaging software.

For western blotting, the 1 cm^2^ square of the IPVL was solubilized in sample buffer and processed for SDS-PAGE.

### Protein extraction and dimethyl labeling

The proteins in the DRM (fractions 2 and 3), as well as fraction 8, were extracted in 10% trichloroacetic acid on ice for 30 min and centrifuged at 20,000 g for 1h. The precipitated pellets were washed with acetone twice.

25 μg protein from the DRM and fraction 8 were reduced with DTT and alkylated by iodoacetamide treatment. Samples were treated with 2.5 μg trypsin overnight and then evaporated to dryness in a speed vacuum centrifuge. Digested samples were reconstituted in 150 μl of 100 mM TEAB. A mixture of 6 μl of 4% CH_2_O and 6 μl of 0.6M NaBH_3_CN was added to the sample solution for the light labeling, and a mixture of 6 μl of 4% ^13^CD_2_O and 6 μl of 0.6M NaBD_3_CN was added in the heavy labeling. Both solutions were incubated for 2 h. The reactions were quenched with 1% ammonia and two differentially labeled samples were acidified with formic acid (FA). They were pooled and applied to solid-phase extraction using the Oasis MCX Cartridge (Waters Corporation, Milford, MA). Samples were dissolved in 0.1% trifluoroacetic acid (TFA) and desalted with the Sep-Pak C18 Vac Cartridge (Waters Corporation).

### NanoLC-MS/MS analysis and quantification

nanoLC-MS/MS analysis was carried out using UltiMate3000 RSLCnano (Dionex, Sunnyvale, CA) coupled to an Orbitrap Fusion (Thermo Fisher Scientific) mass spectrometer equipped with a nanospray Flex Ion Source. The sample was loaded onto an Acclaim PepMap 100 C_18_ trap column (5 μm, 100 μm × 20 mm, 100 Å, Thermo Fisher Scientific) with nanoViper Fittings with 0.5% FA at 20 μL/min for 1.7 min and then separated on an Acclaim PepMap C18 nano column (3 μm, 75μm x 25cm, Thermo Fisher Scientific) and eluted in a 120 min gradient of 5–90% Solvent B. For quality control purposes, a10 fmol injection of standard BSA digest mixture was run as well.

The Orbitrap Fusion operated in positive ion mode with nano spray voltage set at 1.7 kV. The Orbitrap full MS survey scan (m/z 375–1575) was followed by Top 3 second data-dependent Collision Induced Dissociation (CID) MS/MS scans for precursor peptides with 2–7 charges above a threshold ion count of 10,000. All data were acquired by the Xcalibur 3.0 operating software and Orbitrap Fusion Tune Application v. 2.1 (Thermo Fisher Scientific).

### MS data analysis

All MS and MS/MS raw spectra from each sample were searched using Proteome Discoverer v 1.4 (Thermo-Fisher Scientific) using a protein database for *Gallus gallus* downloaded from NCBI on March 15, 2016, with 46,000 entries. The peptide search was performed by two Sequest HT nodes, with the only difference being in static light dimethyl (+28.031Da) and heavy dimethyl (+36.076Da) of any lysine and peptide N-Terminus. The enzyme specificity was set to trypsin with two missed cleavages allowed. The peptide mass tolerance and fragment mass tolerance values were 10 ppm and 0.8 Da, respectively. A fixed carbamidomethyl modification of cysteine and variable methionine oxidation and deamidation of asparagine/glutamine were applied. Identified peptides were filtered for a maximum 1% false discovery rate (FDR) and minimum peptide confidence–high. Proteins were validated based on the presence of two or more unique peptides identified.

For relative quantitation of heavy/light samples, the peak areas of detected precursor ions at each specific m/z corresponding to heavy and light peptides were generated from the precursor ion-based methyl-duplex algorithm in PD 1.4, with a mass tolerance at 4 ppm.

### Statistical analysis

Multiple comparisons were carried out with two-way analysis of variance (ANOVA) and one-way ANOVA followed by Turkey’s HSD test. Pairwise comparisons were performed with a t-test. Results are expressed as means ± SEM. Probability values lower than 0.05 were considered significant.

## Results

### Biochemical characterization of membrane rafts in chicken sperm

**Fig 1:**
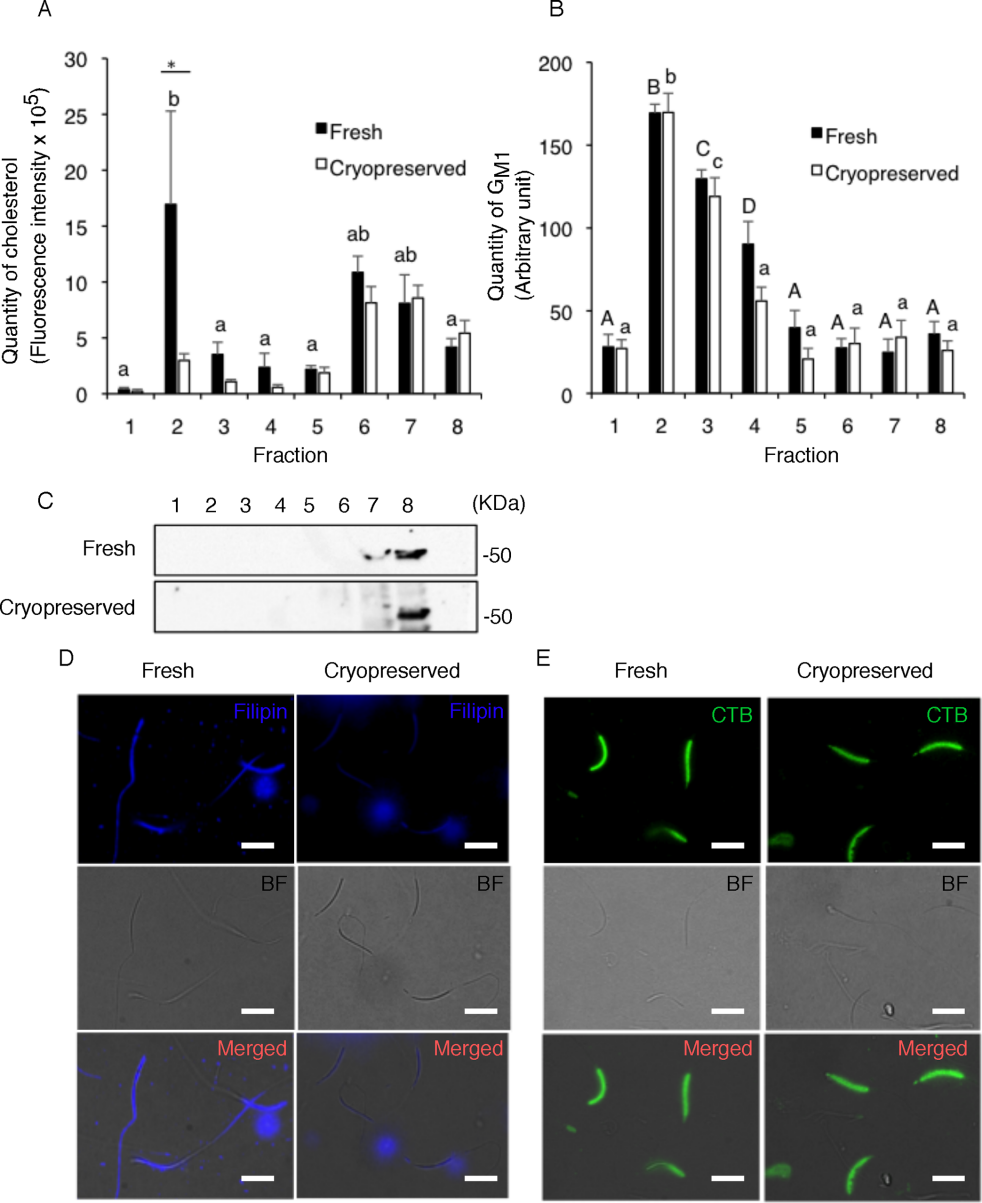
The distribution of lipid contents in the sucrose gradient of DRM isolated from fresh and cryopreserved sperm. Sperm DRM were separated into fractions based on their relative buoyancies. Numbers denote fractions from top (1) to bottom (8) of the tube, with fraction 1 representing the lowest density. Quantification of cholesterol (A) and G_M1_ (B) was performed in the 8 fractions as described. Data are expressed as mean ± SEM (n = 3–6). The different letters denote significant differences between the fractions of the same set (*P* < 0.05). Asterisks denote significant differences between fresh and cryopreserved sperm (*P* < 0.05). Fractions of sperm DRM were processed for SDS-PAGE and subjected to immunoblotting for presence of β-tubulin (C). Fresh and cryopreserved sperm were labeled with Filipin III (D) or CTB-Alexa 488 (E). Images were acquired with a same exposure time between fresh and cryopreserved sperm (n = 3). Bar = 10 μm.

Although membrane rafts have been isolated from mammalian sperm, their biochemical and functional properties in avian sperm have not been characterized. Previously, we found in chicken sperm that cryopreservation induced the loss of cholesterol from the membranes, leading to an early apoptotic event [21, 29]. Considering other studies in culture cells which have demonstrated that cholesterol loss from membrane rafts induced early cellular apoptosis [30–32], cholesterol depletion in cryopreserved sperm is deduced to happen in membrane rafts. However, the compositional changes that occur in membrane rafts remains unknown. To compare the biochemical nature of membrane rafts between fresh and frozen-thawed chicken sperm, the low density DRM was isolated using TX-100 and sucrose density centrifugation, and subjected to cholesterol and G_M1_ quantification. This approach resulted in the formation of an opalescent band at the 5–30% density interface of the gradient in both sperm. This is a characteristic of a low density DRM detected in sperm from several species [11, 33–35]. In fresh sperm, higher cholesterol content was observed in fraction 2 (density; 1.017 g/cm^3^) than the bottom fraction (fraction 8, density; 1.146 g/cm^3^), although there was no difference in cholesterol content among the remaining fractions (Fig 1A). G_M1_ content was higher in fractions 2 and 3 (density; 1.104 g/cm^3^) than in fraction 8 (Fig 1B). Protein content in fraction 2 and 3 was only 7% of total protein separated on sucrose density centrifugation (S1 Fig), consistent with preferential association of a subset of membrane proteins with membrane rafts [36]. Immunoblot analysis revealed that fraction 8 contained a tremendous amount of α-tubulin while others were devoid of it (Fig 1C), suggesting that the bottom fraction mainly consisted of high density non-raft membranes. Taken together, our results suggest that fractions 2 and 3 were representative of the putative DRM and could be categorized into at least 2 types of lipid composition: fraction 2 showed high cholesterol and G_M1_, and fraction 3 showed low cholesterol but high G_M1_. Of note, when sperm were cryopreserved, the sterol content in fraction 2 of the DRM fractions significantly decreased although there was no significant change in other fractions (Fig 1A). However, G_M1_ content did not differ between fresh and cryopreserved sperm throughout fractions (Fig 1B). Consistent with these observations, when the sub-cellular distribution of sterols was examined in fresh sperm using Filipin, strong signal was observed in the plasma membranes with particular intensity in the sperm head region (Fig 1D). However, a feeble signal was observed in cryopreserved sperm. In contrast to this, when the sub-cellular distribution of G_M1_ was detected using CTB-Alexa488, there was no visible difference between fresh and cryopreserved sperm in terms of the localization and intensity of the signal (Fig 1E). These results suggest that cryopreservation induced cholesterol efflux from membrane rafts.

### In vitro binding analysis of membrane raft protein to the IPVL

**Fig 2:**
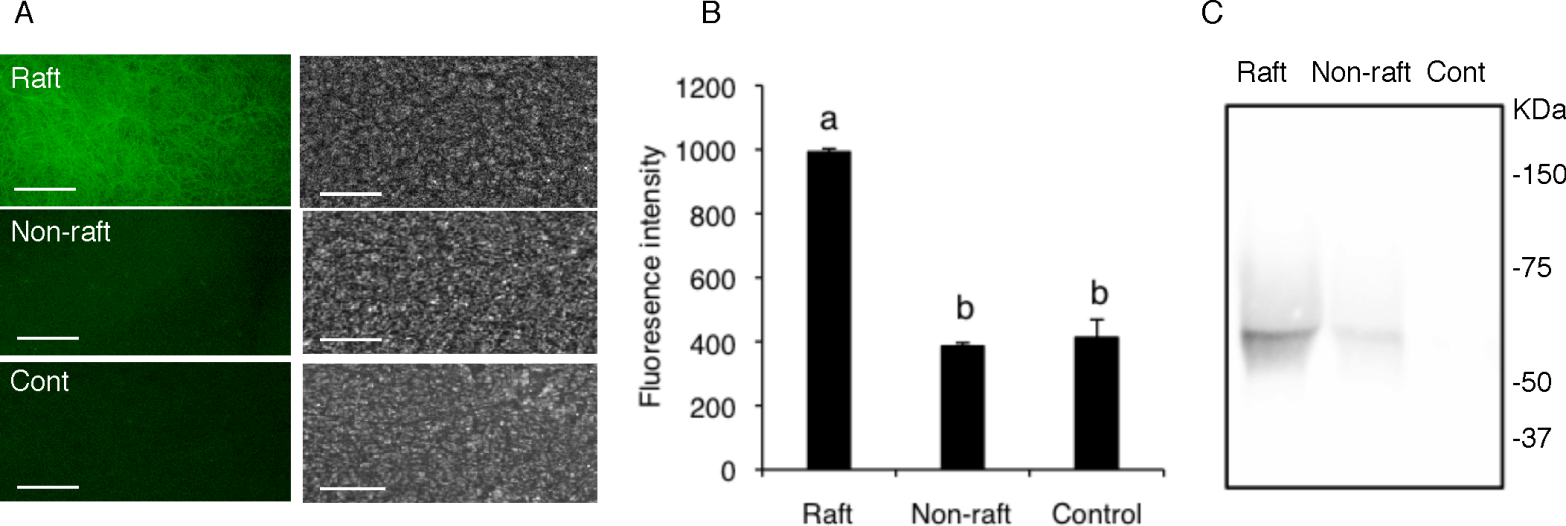
Binding affinity of the low density DRM to the IPVL. The DRM (fraction 2 and 3) and non-raft (fraction 8) proteins were biotinylated and then co-incubated with the IPVL. After washing away unbound proteins, the IPVL was treated with FITC-conjugated streptavidin (A). The binding affinity of the DRM and non-raft proteins were measured by quantification of fluorescence intensity using LAS AF software, as described (B). IPVL binding proteins of the low density DRM and non-raft were purified using the NeutrAvidin-biotin system and were processed for immunoblotting. Data were expressed as mean ± SEM (n = 3). The different letters denote significant difference (*P* < 0.05). Bar = 20 μm.

Our biochemical analyses showed that chicken sperm DRM consists of two different types of lipid composition. Several lines of evidence suggest that a subset of glycolipids and proteins in the DRM fraction possesses the ability to adhere to the ZP [14, 22]. Furthermore, in addition to mammals, DRM associated molecules were found to mediate the binding to the egg in sea urchin [37] and ascidian sperm[38], resulting in the prospect that membrane rafts represent platforms for the organization of molecules involved in the sperm−oocyte interactions [37, 39, 40]. Therefore, we pooled fractions 2 and fraction 3 that displayed distinct lipid composition together and examined the binding affinity of the low density DRM (fractions 2 and 3) and the non-raft membrane fraction (fraction 8) to the IPVL. Interestingly, proteins in the DRM fraction were found to bind to the IPVL more strongly than the non-raft membrane fraction (Fig 2A and 2B). In contrast, the non-raft fraction and control (IPVL labeled with only FITC-streptavidin) failed to bind to the IPVL. To determine the profile of the DRM protein bound to the IPVL, western blot analysis was performed using NeutrAvidin-HRP, showing that the DRM fraction contained a single protein around 60 KDa able to bind to the IPVL while no protein in the non-raft fraction was bound to the IPVL (Fig 2C). These results suggest that membrane rafts are involved in sperm-IPVL interaction in chicken sperm.

### Mass spectrometric analysis for relative quantification of the DRM protein

To facilitate the characterization of the protein composition of the low density DRM fraction, we performed duplex quantitative proteomic analysis using a stable isotope dimethyl labeling approach, which enabled us to identify 258 proteins, including 15 DRM-specific (not identified in non-raft fraction), 67 DRM-enriched (1 < non-raft/DRM), 139 non-raft enriched (1 > non-raft/DRM), and 37 non-raft specific (not identified in DRM fraction) proteins (S1 Table). The set of identified proteins, unique peptides, distinct peptides, and the percentage of proteins with transmembrane domain is summarized in Table 1.

**Table 1:** Quantitative proteomic analysis for relative protein abundance between DRM and non-raft fraction.

Proteins (ratio of non-raft to raft)		Unique Pep^a^	Pep^b^	Tm^c^(%)
Identified proteins	258			
DRM specific	15	30	30	33
DRM enriched (< 1)	67	221	225	40
Non-raft enriched (> 1)	139	472	484	10
Non-raft specific	37	77	77	27

^a^The number of peptide sequences unique to a protein group.

^b^The number of distinct peptide sequences in the protein group.

^c^The presence of transmembrane helices in a protein predicted by SOSUI software (http://bp.nuap.nagoya-u.ac.jp/sosui/).

Of 15 DRM-specific proteins, 14 were shown to be present in sperm by either proteomic analysis or immuno-detection (Table 2). However, the functional roles of these molecules are largely unknown in sperm of any species. Together with this, our results suggest functional regulation of these molecules by membrane rafts in sperm. Furthermore, tectonic-1 has not been identified with regard to their presence in the sperm of any species. Our results of the proteomic comparisons add to the body of knowledge regarding the function of membrane rafts and provide a mechanistic insight into the regulation of sperm function in birds.

**Table 2:** DRM-specific proteins.

Accession	Description	MW	Cov^1^	Pep^2^	Ref^3^
50760928	PREDICTED: CD320 molecule isoform X3	12.5	28.7	2	[41]
971421427	PREDICTED: iron-sulfur cluster assemblyenzyme ISCU, mitochondrial	16.7	17.09	2	[42]
971410655	PREDICTED: protein phosphatase inhibitor 2 isoform X1	23.4	16.51	2	[43]
971444830	PREDICTED: transmembrane protein 120A-like	17.4	14.38	2	[44]
45382787	tetranectin precursor	22.2	11.44	2	[44]
363745270	PREDICTED: cob(I)yrinic acid a,c-diamide adenosyltransferase, mitochondrial isoform X2	25.1	11.21	2	[45]
310750337	proteasome subunit alpha type-4	29.5	9.58	2	[46]
971373773	PREDICTED: prohibitin-2 isoform X1	32.1	7.27	2	[47]
971394300	PREDICTED: heparan-alpha-glucosaminide N-acetyltransferase	68.7	5.65	2	[45]
71895915	GMP reductase 1	37.3	5.51	2	[41]
513210178	PREDICTED: tectonic-1	61.3	4.6	2	-
971410677	PREDICTED: probable cation-transporting ATPase 13A4 isoform X1	108.8	2.94	2	[45]
71895471	acylamino-acid-releasing enzyme	81.2	2.32	2	[48]
971396225	PREDICTED: attractin isoform X3	139.3	2.24	2	[49]
971400272	PREDICTED: cytoplasmic dynein 1 heavy chain	532.8	0.6	2	[42]

^1^Number Sequence coverage (%)

^2^Number of unique peptides

^3^Citations refer to demonstrations of the protein in either testis or sperm

### Characterization of protein profiles

To determine the known molecular functions and biological processes with which the identified proteins are associated, a functional characterization was performed using a PANTHER analysis (S2 Fig)[http://www.pantherdb.org/]. For this analysis, GI accession numbers provided from the NCBI database were converted to each corresponding gene ID manually. Although DRM-enriched, non-raft-enriched, and non-raft specific proteins shared two major functions (binding and catalytic activity), the structural molecule activity was comprised primarily of a large percentage of raft-specific proteins, consistent with a functional role for membrane rafts in scaffolding. Transporter activity was found in all categories, with particular enrichment in DRM-specific and DRM-enriched proteins. Receptor activity was detected in DRM-enriched, non-raft enriched, and non-raft specific proteins, and enzyme regulator activity was shared between non-raft enriched and non-raft specific proteins. Unclassified proteins were the most prevalent in all fractions.

In terms of biological processes, cellular process and metabolic processes were the two major functions across all categories. Although cellular component organization for biogenesis and localization were found in all categories, the later comprised relatively large percentages in DRM-specific and DRM-enriched proteins, suggesting the distinction in protein localization between DRM and non-raft proteins. Unclassified proteins were the most prevalent in all fractions.

## Discussion

In this study, we isolated a low density DRM from chicken sperm to characterize the biochemical composition and functional roles of membrane rafts. Our findings show that membrane rafts play an important role in multiple functions of chicken sperm. This led us to perform proteomic analysis of the relative abundance of DRM to non-raft proteins. These results clearly demonstrated the functional importance of membrane rafts in chicken sperm and provide a foundation to reveal the molecular mechanisms behind sperm functions necessary for fertilization in birds.

To date, the isolation of DRM with TX-100 at low temperature has been widely used for characterization of the compositional and functional nature of membrane rafts in a variety of cell types. This operation was originally generated based on evidence that the profile of partitioned molecules in the liquid-ordered domain of artificial membranes correlates well with its recovery in the DRM fraction [50]. Although caution is warranted when assessing the results of compositional analysis of the DRM fraction [3, 51, 52], this methodology is useful for identifying cellular pathways regulated by the organization of membrane rafts [53]. In the current study, we characterized both lipid and protein composition in the low density DRM and non-raft fractions separated on a density gradient. The result of quantitative proteomic comparisons agreed with the notion that only a subset of proteins identified in the low density DRM was enriched in DRM relative to the non-raft fraction and thus suggest the functional distinction between membrane domains in chicken sperm.

Our quantification of cholesterol revealed that cholesterol levels are specifically decreased in the low density DRM fraction following cryopreservation. Similarly, the localization of sterols using Filipin showed a sharp decrease in sterol content in the membranes of cryopreserved sperm. In accordance with our previous findings that cryopreservation-induced sterol loss causes an early apoptotic events [21], resulting in a decline of fertilizing ability in chicken sperm [29], the results of present study suggest that a compositional rearrangement of membrane rafts specifically occurs in response to cryopreservation-induced sterol loss, leading to the functional damage in chicken sperm via an induction of an early apoptosis. However, G_M1_ quantification showed no difference in the low density DRM, although there was a tendency for decreasing the amount in the fraction with a lower buoyant density, such as in fractions 4 and 5. There would be a possibility to explain this phenomenon. Firstly, this might represent the heterogeneity of membrane rafts. Because DRM association of proteins and lipids reflects only the end result of the extraction process, it has been suggested that the association does not necessarily manifest in the same domains in living cells [36]. Previous studies demonstrated in T lymphocytes that several proteins and sphingolipids localize into different membrane domains of the cells even though they are all associated with the same DRM fraction [54]. Therefore, our results suggest the possibility that chicken sperm possesses multiple membrane raft sub-types each with a different lipid composition. In support of this conclusion, we previously reported in murine sperm that membrane rafts consist of at least three different sub-types with varying lipid and protein composition [24]. Thus, it is possible to assume that the G_M1_-enriched DRM fraction might be derived from both cholesterol-independent and cholesterol-dependent membrane rafts. Studies using a small intestinal brush border membrane have shown that cholesterol is dispensable for the formation of the DRM when a high concentration of sphingolipids is present in the membranes [55], which also supports a previous study that modeled liposomal membranes [56]. These previous results, combined with our finding that a tremendous amount of G_M1_ was present in chicken sperm DRM, suggest that chicken sperm possesses stable G_M1_-enriched membrane rafts. Further study will be required to determine the roles of these G_M1_-enriched domains.

In this study, we found that the chicken sperm DRM proteins possessed a high affinity for binding to the IPVL. Together with previous studies of the functional involvement of membrane rafts in sperm−oocyte interaction in vertebrates [14, 22, 38]and invertebrates [37], our data corroborates the possibility that the role of membrane rafts in sperm−egg binding is conserved across phyla. Earlier biochemical studies in Japanese quail demonstrated that a 45 KDa sperm acrosin plays an important role in the binding of sperm to the IPVL during fertilization [18]. Therefore, we examined which proteins in chicken sperm DRM were capable of binding to the IPVL. Interestingly, our results showed that a 60 kDa single protein enriched in the DRM relative to the non-raft fraction was able to bind to the IPVL. Although several mammailan sperm proteins were found to mediate binding to the ZP to compensate for losses of function [57, 58], only acrosin has been shown to be responsible for sperm binding to the IPVL in birds [18]. This motivated us to perform immunoblotting for the presence of acrosin in chicken sperm DRM. We found that acrosin was absent from the DRM but was abundant in the non-raft fraction (S3 Fig), suggesting the emergence of a new candidate molecule that mediates the binding of sperm to the IPVL in birds. In fact, a literature search of proteomic characterization of mammalian sperm rafts showed that PH-20, basigin, and the cysteine-rich secretory protein 1, which are known to be involved in the sperm binding to the ZP, also are associated with membrane rafts [11, 24, 59]. In addition, glioma pathogenesis-related 1-like protein 1, which plays a role in sperm-egg interaction, is enriched in bull sperm DRM [60]. Although it cannot be ruled out that species differences might exist, our results provide a potential foundation for revealing the complexity of redundant mechanisms involved in sperm-egg recognition in avian species.

Quantitative mass spectrometry has been used to compare the complex profiles and relative abundance of proteins between different membrane domains [61]. Using quantitative proteomic analysis combined with stable isotope dimethyl-labeling approaches in chicken sperm, we found that 82 proteins were either exclusively or relatively more highly associated with the DRM than other regions. In addition, it is intriguing that we found enrichment of a variety of ion transporters/channels in the DRM. For example, plasma membrane Ca^2+^ ATPase (PMCA) 1 and 4 were prominently associated with the DRM. In murine sperm, the PMCA family plays a primary role in the maintenance of cytosolic Ca_2_^+^ concentration which is important for induction of the acrosome reaction and hyperactivated motility [62]. Previous studies in murine sperm revealed that PMCA4 is highly expressed and localized to the APM and the principal piece of the tail, suggesting the involvement of PMCA4 with multiple functions in sperm [63, 64]. In fact, the DRM association of PMCA4 was reported by previous proteomic studies performed with murine and human sperm [14, 23]. In addition, functional characterization of the bull sperm membrane rafts demonstrated that PMCA4 is present in membrane rafts and its activity appears to be regulated by membrane raft-enriched lipids [65]. Taken together, our results suggest the functional importance of PMCA4 in chicken sperm.

Previously, sodium/potassium transport of ATPase (Na+/K+-ATPase) activity was shown to play a role in the induction of the acrosome reaction, the maintenance of flagellar motility, and fertilizing ability in mammalian sperm [66–69]. It is noteworthy that we found that multiple subunits (α1, α3, β1) of Na^+^/K^+^-ATPase is enriched in the DRM with the close relative abundance to those in the non-raft fraction. Considering that α1 and β1 subunits assemble the complex of Na^+^/K^+^-ATPase [70], this result supports the accuracy of the proteomic analysis in the current study. Previous localization experiments performed in bovine sperm demonstrated that α1 and β1 subunits are localized to the APM, while α3 subunit is present in both the APM and post-acrosomal plasma membranes [71]. An electrophysiological study in motoneurons demonstrated the difference in localization and affinities for Na^+^/K^+^-ATPase between α1 and α3 subunits [72], suggesting the functional discrimination of these subunits. Therefore, these results suggest that investigations of the functional roles of these subunits in sperm will be of great interest.

In this study, we found that some of mitochondrial proteins were relatively abundant in DRM fraction to non-raft fraction. In fact, the presence of membrane rafts in mitochondrial is controversial in somatic cells. Because previous quantitative proteomic study of membrane rafts isolated from cultured cells suggested that mitochondrial proteins could be contaminants that are simply co-isolated with raft domains during separation process[73]. However, there are several reports suggesting that intracellular organelles such as mitochondrial might possess membrane rafts in somatic cells[74–76]. In sperm, multiple proteomic studies for characterization of membrane rafts identified mitochondrial proteins associated with membrane rafts. One possibility resulting in this discrepancy is difference in mitochondrial membrane between cell types[14, 22, 59]. Sperm are well known to possess mitochondria that differs from somatic cells in morphology, localization, interaction with the membranes of other sub-cellular compartments. Furthermore, recent studies demonstrated in somatic cells that mitochondrial membranes possess raft-like microdomains formed by membrane scrambling between endoplasmic reticulum and mitochondria [77]. Therefore, our results, combined with previous proteome profiling of sperm membrane rafts, suggest a potential investigation for compositional and structural nature of mitochondrial membranes in sperm.

In summary, our results provide compelling evidence that membrane rafts play important roles in a variety of cellular processes in chicken sperm by restricting the functional molecules to the membrane domains. Biochemical and cell biological analysis of the DRM demonstrated that membrane rafts appear to be involved in several important functions, including mechanisms involved in the impairment of post-thaw fertility after cryopreservation and the binding of sperm to the IPVL. Quantitative proteomic comparison for estimating the abundance of DRM relative to non-raft proteins identified a total 258 proteins. Our results indicate that there are significant functional distinctions between membrane domains, providing a foundation for further investigation involving the cellular and molecular basis for the regulation of sperm functions in birds.

## Supporting information

S1 Fig Protein contents in membrane fractions. Sperm membranes were isolated under presence of 1% TX-100, and separated into 8 fractions following by sucrose density gradient centrifugation. Protein amount was quantified by micro BCA assay (n = 4). ^a-d^P < 0.05.
(PDF)

S2 Fig Molecular function and biological process associated with DRM. (PDF)

S3 Fig Immuno-detection of acrosin. Protein of low density DRM (fractions 2 and 3) and non-raft (fraction 8) were extracted with 4% trichloroaceticacid on ice, and subjected to immunoblotting for the presence of acrosin. Sperm (2x10^7^) were utilized as a control. Immuno-reactivity in sperm was found at predicted molecular weight. No acrosin was detected in DRM although it was found in non-raft.
(PDF)

S1 Table Proteome comparison for relative protein abundance between DRM and non-raft. (PDF)
