# Supplementary figures and images for: Characterization of the functions and proteomes associated with membrane rafts in chicken sperm
Source: PLoS One. 2017 Nov 2;12(11):e0186482. doi: 10.1371/journal.pone.0186482 (PMC5667776; doi:10.1371/journal.pone.0186482)

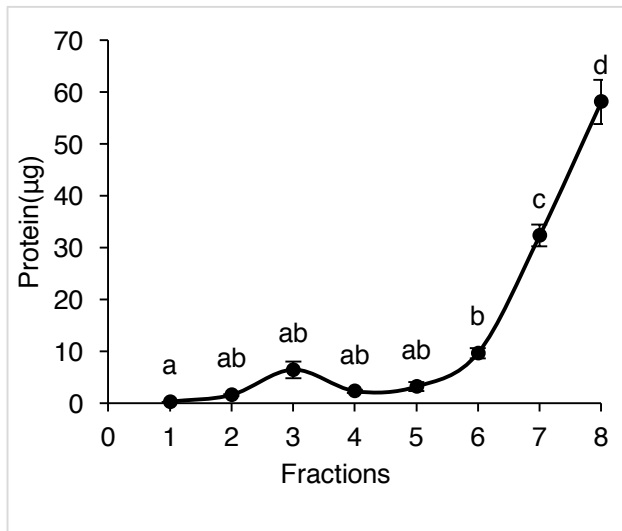

Supplement: S1 Fig — Sperm membranes were isolated under presence of 1% TX-100, and separated into 8 fractions following by sucrose density gradient centrifugation. Protein amount was quantified by micro BCA assay (n = 4). a-dP < 0.05. (PDF) [file pone.0186482.s001.pdf]

S3 Fig

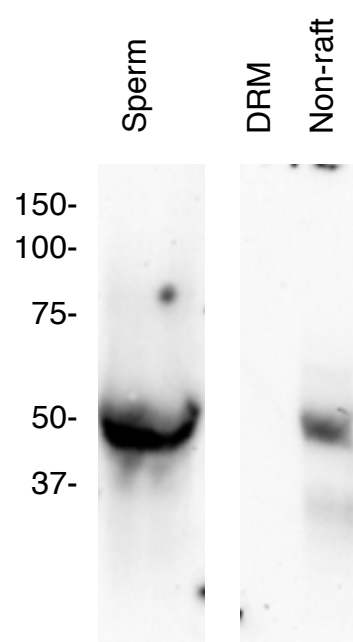

Supplement: S3 Fig — Sperm (2x10^7^) were utilized as a control. Immuno-reactivity in sperm was found at predicted molecular weight. No acrosin was detected in DRM although it was found in non-raft. (PDF) [file pone.0186482.s003.pdf]
