# Supplementary material for: Characterization of the functions and proteomes associated with membrane rafts in chicken sperm
Source: PLoS One. 2017 Nov 2;12(11):e0186482. doi: 10.1371/journal.pone.0186482 (PMC5667776; doi:10.1371/journal.pone.0186482)
Supplement: S2 Fig — (PDF) [file pone.0186482.s002.pdf]

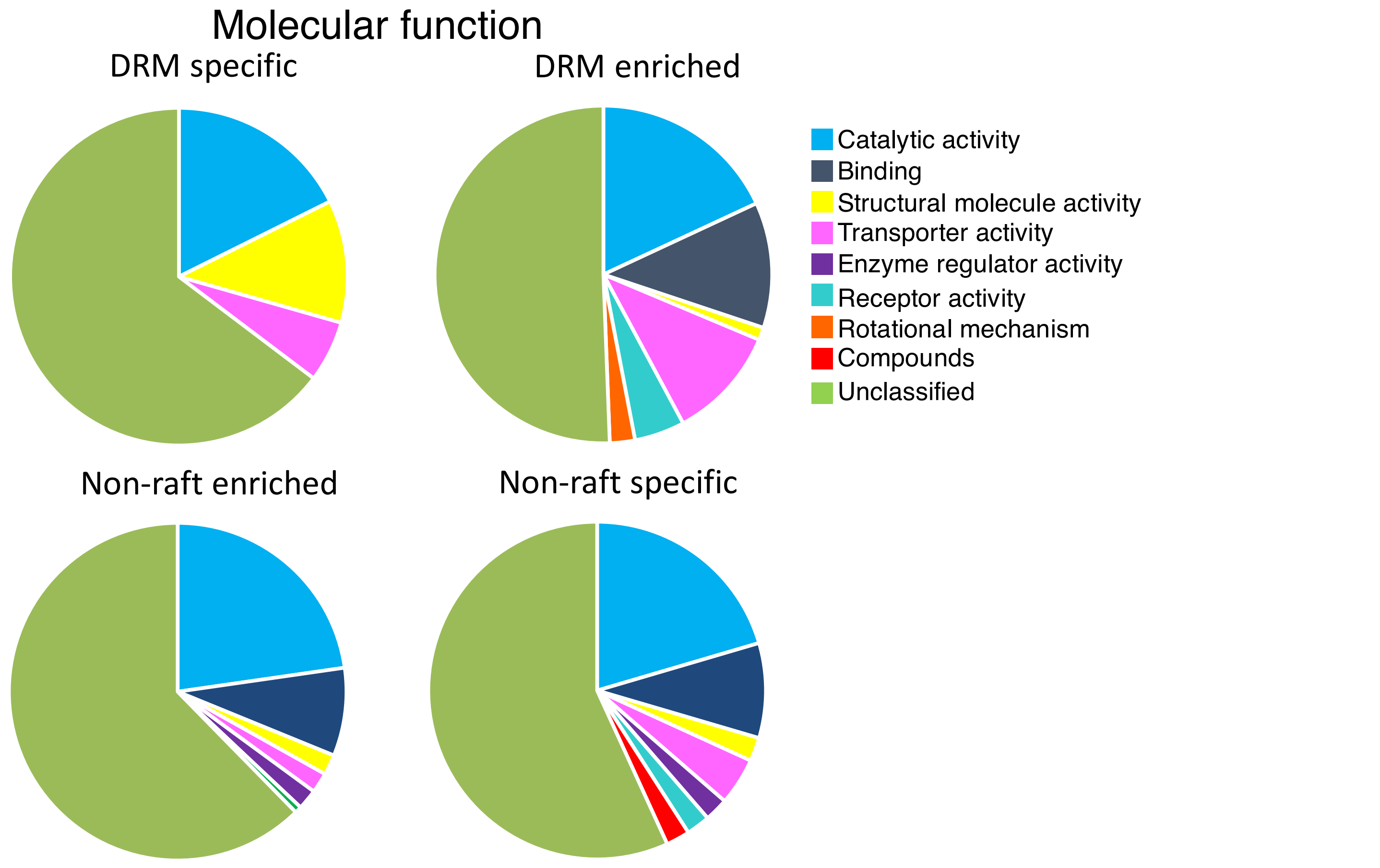
Molecular function
DRM specific
DRM enriched
Catalytic activity
Binding
Structural molecule activity
Transporter activity
Enzyme regulator activity
Receptor activity
Rotational mechanism
Compounds
Unclassified
Non-raft enriched
Non-raft specific

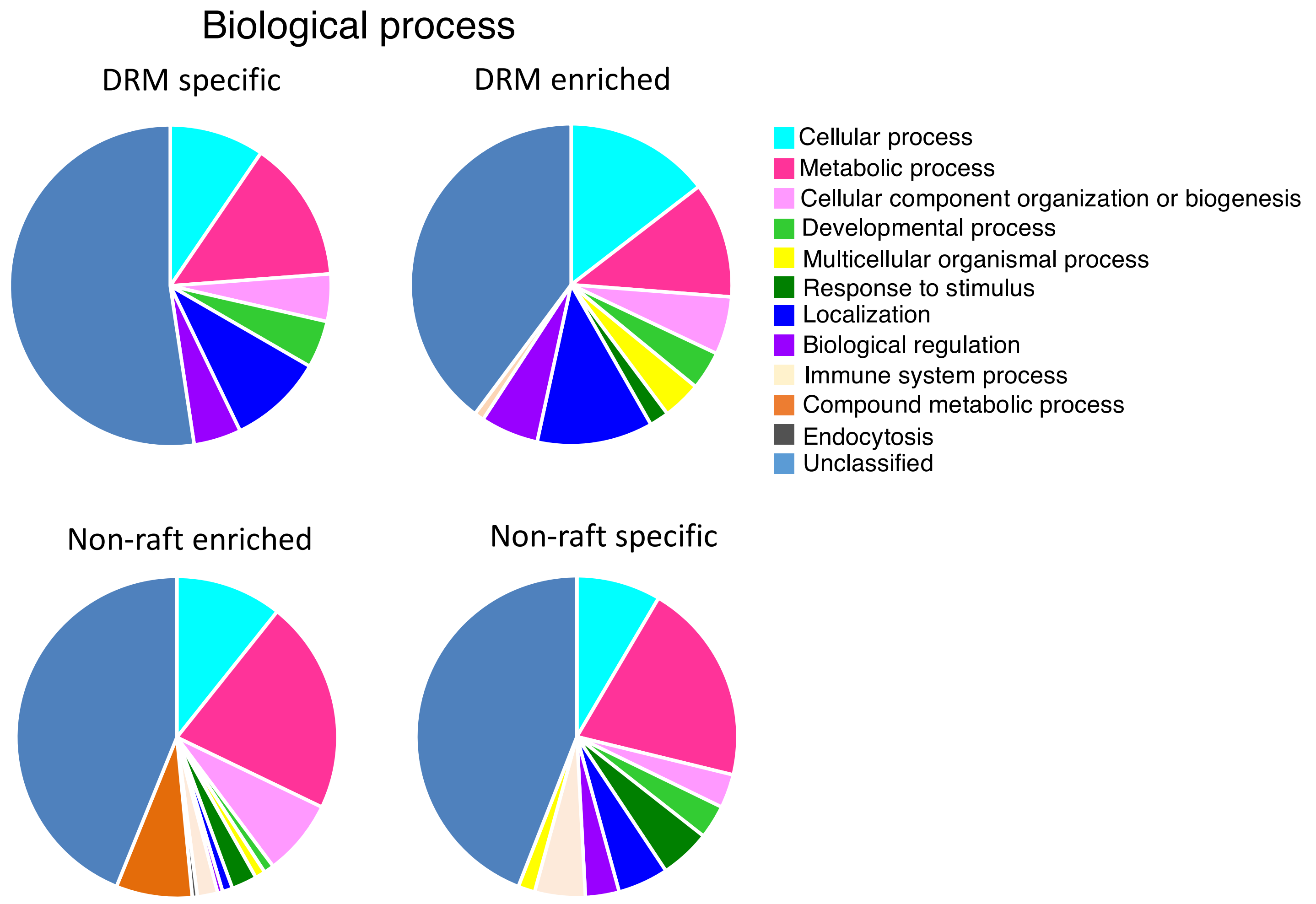
Biological process
DRM specific
DRM enriched
Cellular process
Metabolic process
Cellular component organization or biogenesis
Developmental process
Multicellular organismal process
Response to stimulus
Localization
Biological regulation
Immune system process
Compound metabolic process
Endocytosis
Unclassified
Non-raft enriched
Non-raft specific
