# Supplementary material for: Characterization of the functions and proteomes associated with membrane rafts in chicken sperm
Source: PLoS One. 2017 Nov 2;12(11):e0186482. doi: 10.1371/journal.pone.0186482 (PMC5667776; doi:10.1371/journal.pone.0186482)
Supplement: S1 Table — (PDF) [file pone.0186482.s004.pdf]

S1 Table

A. DRM specific proteins

Accession	Description	MW	Coverage	Pep[1]	Ratio[2]	TM[3]
50760928	PREDICTED: CD320 molecule isoform X3	12.5	28.70	2	-	-
971421427	PREDICTED: iron-sulfur cluster assembly enzyme ISCU, mitochondrial	16.7	17.09	2	-	-
971410655	PREDICTED: protein phosphatase inhibitor 2 isoform X1	23.4	16.51	2	-	-
971444830	PREDICTED: transmembrane protein 120A-like	17.4	14.38	2	-	-
45382787	tetranectin precursor	22.2	11.44	2	-	1
363745270	PREDICTED: cob(I)yrinic acid a,c-diamide adenosyltransferase, mitochondrial isoform X2	25.1	11.21	2	-	-
310750337	proteasome subunit alpha type-4	29.5	9.58	2	-	-
971373773	PREDICTED: prohibitin-2 isoform X1	32.1	7.27	2	-	-
971394300	PREDICTED: heparan-alpha-glucosaminide N-acetyltransferase	68.7	5.65	2	-	10
71895915	GMP reductase 1	37.3	5.51	2	-	-
513210178	PREDICTED: tectonic-1	61.3	4.60	2	-	1
971410677	PREDICTED: probable cation-transporting ATPase 13A4 isoform X1	108.8	2.94	2	-	7
71895471	acylamino-acid-releasing enzyme	81.2	2.32	2	-	-
971396225	PREDICTED: attractin isoform X3	139.3	2.24	2	-	1
971400272	PREDICTED: cytoplasmic dynein 1 heavy chain 1	532.8	0.60	2	-	-

B. DRM-enriched proteins

Accession	Description	MW	Coverage	Pep[1]	Ratio[2]	TM[3]
118099442	PREDICTED: erythrocyte band 7 integral membrane protein	30.8	16.73	3	0.010	1
971378251	PREDICTED: phospholipid-transporting ATPase IB isoform X3	129.3	2.79	3	0.012	10
971398904	PREDICTED: disintegrin and metalloproteinase domain-containing protein 21-like isoform X2	68.0	5.12	3	0.013	1
971401669	PREDICTED: voltage-dependent anion-selective channel protein 2 isoform X1	31.5	11.82	3	0.013	-

971438272	PREDICTED: NAD(P) transhydrogenase, mitochondrial isoform X2	110.6	7.38	7	0.013	13
971430011	PREDICTED: disintegrin and metalloproteinase domain-containing protein 32-like	80.5	5.55	3	0.014	2
971382925	PREDICTED: NADH dehydrogenase [ubiquinone] iron-sulfur protein 6, mitochondrial isoform X2	11.7	30.84	2	0.014	-
513157871	PREDICTED: pendrin isoform X2	70.7	5.12	3	0.018	10
75571417	RecName: Full=Cytochrome b-c1 complex subunit Rieske, mitochondrial	29.4	14.71	3	0.020	-
75571379	RecName: Full=Vesicle-associated membrane protein 7	24.7	10.91	2	0.022	2
971411244	PREDICTED: phospholipid scramblase 1	33.6	7.54	2	0.022	-
45383430	solute carrier family 15 member 1	79.3	3.92	2	0.023	-
971433268	PREDICTED: testis anion transporter 1 isoform X4	91.5	6.23	4	0.024	13
71896123	NADH dehydrogenase [ubiquinone] flavoprotein 1, mitochondrial	50.1	5.45	2	0.025	11
363729602	PREDICTED: disintegrin and metalloproteinase domain-containing protein 9-like isoform X2	83.4	4.61	2	0.025	-
45383628	guanine nucleotide-binding protein G(k) subunit alpha	40.4	5.93	2	0.029	2
57529753	NADH-ubiquinone oxidoreductase 75 kDa subunit, mitochondrial	79.5	8.38	5	0.035	-
971448224	PREDICTED: mitochondrial 2-oxoglutarate/malate carrier protein, partial	27.8	11.86	2	0.036	-
121759	RecName: Full=Solute carrier family 2, facilitated glucose transporter member 3	54.1	10.48	5	0.037	-
75571296	RecName: Full=Vesicle-trafficking protein SEC22b	24.7	13.49	2	0.037	11
906848897	Chain O, Cytochrome Bc1 Complex From Chicken With Designed Inhibitor Bound	46.6	16.55	7	0.041	-
971421200	PREDICTED: gamma-glutamyltranspeptidase 1 isoform X2	61.5	16.52	8	0.045	2

2500201	RecName: Full=Cell division control protein 42 homolog	21.3	10.99	2	0.046	-
513161834	PREDICTED: mitochondrial import receptor subunit TOM70	72.5	5.36	3	0.052	1
971380453	PREDICTED: ras-related protein Ral-A	23.5	12.14	2	0.060	-
57530014	ras-related protein R-Ras2	23.2	12.32	2	0.062	-
513175173	PREDICTED: neutral and basic amino acid transport protein rBAT	77.1	3.98	3	0.066	1
971433783	PREDICTED: plasma membrane calcium-transporting ATPase 4 isoform X5	128.0	6.00	5	0.068	8
363743079	PREDICTED: ATP synthase F(0) complex subunit B1, mitochondrial	28.6	12.16	3	0.074	-
60099193	hypothetical protein RCJMB04_33k20	48.9	4.97	2	0.077	-
114372	RecName: Full=Sodium/potassium-transporting ATPase subunit alpha-1	112.2	11.56	9	0.078	8
114391	RecName: Full=Sodium/potassium-transporting ATPase subunit beta-1	34.9	6.89	2	0.079	1
971370161	PREDICTED: ATP synthase subunit gamma, mitochondrial isoform X1	27.7	8.37	2	0.079	-
114380	RecName: Full=Sodium/potassium-transporting ATPase subunit alpha-3	111.2	6.63	4	0.083	7
971372403	PREDICTED: cytoskeleton-associated protein 4	62.4	6.91	3	0.084	1
347361001	vesicle-fusing ATPase	82.1	3.24	2	0.085	-
971371017	PREDICTED: ras-related protein Rap-1b isoform X1	20.8	13.04	2	0.085	-
513162510	PREDICTED: ATP synthase-coupling factor 6, mitochondrial	12.4	25.23	3	0.086	-
3122072	RecName: Full=Elongation factor 1-alpha 1; Short=EF-1-alpha-1	50.1	6.06	3	0.087	-
118099967	PREDICTED: ATP synthase subunit d, mitochondrial	18.3	10.56	2	0.092	-
971420099	PREDICTED: STIP1 homology and U box-containing protein 1 isoform X1	33.3	11.19	2	0.092	-
513162756	PREDICTED: ATP synthase subunit O, mitochondrial	26.6	12.45	3	0.092	-

3851616	succinate dehydrogenase Fp subunit, partial	54.9	10.02	4	0.103	-
513161533	PREDICTED: fatty acid amide hydrolase-like	71.1	4.73	2	0.105	1
52138713	neprilysin	85.3	4.13	2	0.108	1
22775582	ATP/ADP antiporter [Gallus gallus]	32.8	8.72	3	0.130	-
971446371	PREDICTED: stomatin-like protein 2, mitochondrial	33.3	7.26	2	0.141	-
971437277	PREDICTED: ATP synthase subunit alpha, mitochondrial isoform X3	54.8	15.22	7	0.141	-
448261627	ATP synthase subunit beta, mitochondrial precursor	56.6	15.20	6	0.145	-
971412546	PREDICTED: secretory carrier-associated membrane protein 2	36.9	8.11	2	0.149	4
82081935	RecName: Full=LETM1 and EF-hand domain-containing protein 1, mitochondrial	85.8	4.65	3	0.169	1
971429939	PREDICTED: uncharacterized protein LOC100859804	166.4	6.15	7	0.174	3
462065	RecName: Full=Fatty acid-binding protein, brain	14.9	12.12	2	0.213	-
971377343	PREDICTED: spermatid-associated protein isoform X1	38.2	6.40	2	0.232	-
513162786	PREDICTED: chloride intracellular channel protein 6	71.1	5.15	2	0.255	-
71897249	potassium-transporting ATPase alpha chain 2	114.3	7.74	5	0.273	5
53127818	hypothetical protein RCJMB04_3p21, partial	22.0	23.20	4	0.278	-
971437594	PREDICTED: ras GTPase-activating-like protein IQGAP2 isoform X7	164.1	1.54	2	0.284	-
1020104	melanotransferrin/EOS47	80.9	3.52	2	0.343	-
364505923	Chain L, Remodeling Of Actin Filaments By Adf Cofilin Proteins	41.6	7.49	3	0.394	-
971411987	PREDICTED: pyruvate kinase PKM isoform X1	57.8	18.49	9	0.756	-
61097999	saccharopine dehydrogenase-like oxidoreductase	46.8	5.99	2	0.788	-
82245348	RecName: Full=Ras-related protein Rab-2A	23.5	24.06	3	0.803	-
135464	RecName: Full=Tubulin beta-3 chain	49.8	13.48	5	0.905	-
85544005	Chain B, Avian Respiratory Complex Ii With 3-nitropropionate And Ubiquinone	28.6	15.08	3	0.929	-
971384738	PREDICTED: dihydropyrimidinase isoform X1	51.2	7.64	3	0.975	-

513163546	PREDICTED: serine/threonine-protein phosphatase with EF-hands 1 isoform X2	65.0	5.36	2	0.997	1

C. Non-raft enriched proteins

Accession	Description	MW	Coverage	Pep[1]	Ratio[2]	TM[3]
119331154	profilin-2	15.0	11.43	2	1.006	-
971412469	PREDICTED: vacuolar protein sorting-associated protein 13C isoform X3	406.9	1.10	3	1.012	-
971449598	PREDICTED: coiled-coil domain-containing protein 70, partial	90.8	2.94	2	1.089	-
300659448	unnamed protein product	58.0	8.75	4	1.326	-
971451287	PREDICTED: profilin-1-like	16.0	28.29	3	1.440	-
513176157	PREDICTED: cAMP and cAMP-inhibited cGMP 3',5'-cyclic phosphodiesterase 10A isoform X5	89.5	4.69	3	1.453	1
82233783	RecName: Full=Peroxiredoxin-6	25.0	13.39	2	1.532	-
767172158	T-complex protein 1 subunit eta	59.4	3.49	2	1.573	-
113206112	transitional endoplasmic reticulum ATPase	89.3	2.73	2	1.609	-
2494246	RecName: Full=Elongation factor 2	95.3	5.01	4	1.670	-
971435910	PREDICTED: long-chain-fatty-acid--CoA ligase ACSBG2 isoform X4	75.9	16.20	9	1.695	-
53127394	hypothetical protein RCJMB04_2c6, partial	24.3	11.84	2	1.740	1
118097631	PREDICTED: heat shock 70 kDa protein 4 isoform X1	94.2	2.98	2	1.753	-
60302718	T-complex protein 1 subunit beta	57.4	6.92	3	1.758	-
82197879	RecName: Full=Na(+)/H(+) exchange regulatory cofactor NHE-RF1	35.8	7.81	2	1.784	-
14278286	Chain B, Crystal Structure Of Avian Atic, A Bifunctional Transformylase And Cyclohydrolase Enzyme In Purine Biosynthesis At 1.75 Ang. Resolution	64.2	7.42	3	1.831	-
971431223	PREDICTED: adenylyl cyclase-associated protein 1 isoform X3	51.0	13.65	3	1.876	-
363745941	PREDICTED: prefoldin subunit 5	17.2	10.97	2	1.889	-
971432201	PREDICTED: LOW QUALITY PROTEIN: T-complex protein 1 subunit gamma	60.4	6.06	3	1.925	-

82233762	RecName: Full=T-complex protein 1 subunit zeta	57.6	6.04	3	1.957	-
71895853	electron transfer flavoprotein-ubiquinone oxidoreductase, mitochondrial	53.1	7.13	3	2.087	-
82085819	RecName: Full=T-complex protein 1 subunit theta	59.4	13.14	6	2.130	-
57530301	T-complex protein 1 subunit alpha	60.4	8.96	4	2.164	-
971380294	PREDICTED: ATP-dependent 6-phosphofructokinase, platelet type isoform X3	86.0	8.42	5	2.375	-
82190481	RecName: Full=Heat shock cognate 71 kDa protein	70.8	13.47	3	2.377	-
60302774	T-complex protein 1 subunit epsilon	59.7	3.88	2	2.602	-
3122036	RecName: Full=Dihydropyrimidinase-related protein 2	62.3	11.36	5	2.813	-
330417943	fructose-bisphosphate aldolase C	39.3	27.20	8	3.141	-
513222537	PREDICTED: dihydrolipoyllysine-residue acetyltransferase component of pyruvate dehydrogenase complex, mitochondrial	66.5	4.43	3	3.142	-
971374025	PREDICTED: sperm-associated antigen 17	251.0	2.18	3	3.211	-
82197897	RecName: Full=Stress-70 protein, mitochondrial	73.1	9.63	5	3.389	-
123586	RecName: Full=Heat shock 70 kDa protein	69.7	18.93	5	3.409	-
157954047	heat shock protein HSP 90-alpha	84.0	16.76	10	3.491	-
482845617	aldo-keto reductase family 1, member B10 (aldose reductase)	35.6	6.01	2	3.499	-
14278286	Chain B, Crystal Structure Of Avian Atic, A Bifunctional Transformylase And Cyclohydrolase Enzyme In Purine Biosynthesis At 1.75 Ang. Resolution	64.2	7.42	3	1.831	-
971431223	PREDICTED: adenylyl cyclase-associated protein 1 isoform X3	51.0	13.65	3	1.876	-
363745941	PREDICTED: prefoldin subunit 5	17.2	10.97	2	1.889	-
971432201	PREDICTED: LOW QUALITY PROTEIN: T-complex protein 1 subunit gamma	60.4	6.06	3	1.925	-
82233762	RecName: Full=T-complex protein 1 subunit zeta	57.6	6.04	3	1.957	-
71895853	electron transfer flavoprotein-ubiquinone oxidoreductase, mitochondrial	53.1	7.13	3	2.087	-

82085819	RecName: Full=T-complex protein 1 subunit theta	59.4	13.14	6	2.130	-
57530301	T-complex protein 1 subunit alpha	60.4	8.96	4	2.164	-
971380294	PREDICTED: ATP-dependent 6-phosphofructokinase, platelet type isoform X3	86.0	8.42	5	2.375	-
82190481	RecName: Full=Heat shock cognate 71 kDa protein	70.8	13.47	3	2.377	-
60302774	T-complex protein 1 subunit epsilon	59.7	3.88	2	2.602	-
3122036	RecName: Full=Dihydropyrimidinase-related protein 2	62.3	11.36	5	2.813	-
330417943	fructose-bisphosphate aldolase C	39.3	27.20	8	3.141	-
513222537	PREDICTED: dihydrolipoyllysine-residue acetyltransferase component of pyruvate dehydrogenase complex, mitochondrial	66.5	4.43	3	3.142	-
971374025	PREDICTED: sperm-associated antigen 17	251.0	2.18	3	3.211	-
82197897	RecName: Full=Stress-70 protein, mitochondrial	73.1	9.63	5	3.389	-
123586	RecName: Full=Heat shock 70 kDa protein	69.7	18.93	5	3.409	-
157954047	heat shock protein HSP 90-alpha	84.0	16.76	10	3.491	-
482845617	aldo-keto reductase family 1, member B10 (aldose reductase)	35.6	6.01	2	3.499	-
57525126	heat shock protein 75 kDa, mitochondrial	79.6	3.69	2	3.814	-
118083310	PREDICTED: ubiquitin carboxyl-terminal hydrolase 5 isoform X2	93.2	4.09	2	4.041	-
194220334	heat shock protein 108	91.2	3.16	2	4.091	1
971371983	PREDICTED: aldose reductase-like	35.6	7.64	2	4.300	-
971390685	PREDICTED: trifunctional enzyme subunit alpha, mitochondrial isoform X1	81.3	3.85	2	4.377	-
971398444	PREDICTED: leucine-rich repeat-containing protein 57 isoform X1	26.7	7.98	2	4.472	-
363730269	PREDICTED: thioredoxin domain-containing protein 3	66.2	3.80	2	4.561	-
758818524	astacin-like metalloendopeptidase precursor	46.4	5.38	2	4.739	1
60302800	heat shock 70 kDa protein 4L	94.8	5.93	3	4.808	-
363744876	PREDICTED: hydroxysteroid dehydrogenase-like protein 2	44.2	5.08	2	4.856	-

971412156	PREDICTED: isocitrate dehydrogenase [NAD] subunit alpha, mitochondrial isoform X2	38.9	6.15	2	5.148	-
971397022	PREDICTED: ovochymase-2 isoform X4	84.0	3.47	2	5.313	1
971415983	PREDICTED: acyl-CoA dehydrogenase family member 9, mitochondrial isoform X1	56.5	7.20	3	5.352	-
309319792	V-type proton ATPase subunit H	55.4	5.83	2	5.472	-
61098372	60 kDa heat shock protein, mitochondrial precursor	60.9	10.12	4	5.472	-
63880	unnamed protein product, partial	96.3	4.76	3	5.546	-
971382475	PREDICTED: ribosyldihydronicotinamide dehydrogenase [quinone] isoform X3	22.2	10.77	2	5.668	-
971371289	PREDICTED: cullin-associated NEDD8-dissociated protein 1	136.3	2.93	4	5.869	-
1730518	RecName: Full=Phosphoglycerate kinase	44.7	12.71	4	5.964	-
2506442	RecName: Full=Glyceraldehyde-3-phosphate dehydrogenase	35.7	17.12	5	6.646	-
118087908	PREDICTED: serine/threonine-protein phosphatase 2A 56 kDa regulatory subunit alpha isoform	55.2	6.09	2	6.752	-
363742258	PREDICTED: C-Myc-binding protein	11.9	19.42	2	6.800	-
310750374	pyruvate dehydrogenase E1 component subunit beta, mitochondrial	38.9	14.76	5	7.638	-
34921412	RecName: Full=Gelsolin; AltName: Full=Actin-depolymerizing factor	85.8	3.98	3	7.712	2
239586402	glycinamide ribonucleotide synthetase, partial	39.0	6.79	2	7.807	-
971377183	PREDICTED: importin-5	123.7	7.73	7	8.123	-
61098338	dihydrolipoyllysine-residue succinyltransferase component of 2-oxoglutarate dehydrogenase complex, mitochondrial	49.3	3.47	2	8.411	-
4033392	RecName: Full=78 kDa glucose-regulated protein	72.0	14.88	6	8.470	-
532524932	ES1 protein homolog, mitochondrial-like	23.9	14.67	3	8.687	-
770075452	electron transfer flavoprotein subunit alpha, mitochondrial	35.3	11.08	2	8.965	-
45384462	V-type proton ATPase catalytic subunit A	68.4	3.93	2	9.020	-
60302740	pyruvate dehydrogenase E1 component subunit alpha, somatic form, mitochondrial precursor	44.4	6.52	2	9.199	-

5705960	immunoglobulin alpha heavy chain	61.5	8.21	4	9.201	-
45384392	apolipoprotein A-IV precursor	40.8	16.94	5	9.300	
971418042	PREDICTED: hexokinase-2-like isoform X2	103.3	10.74	9	9.532	-
82197810	RecName: Full=UMP-CMP kinase	22.2	9.18	2	9.591	-
21730886	Chain D, Crystal Structure Of Native Chicken Fibrinogen With Two Different Bound Ligands	54.1	5.91	2	9.747	-
971435866	PREDICTED: lon protease homolog, mitochondrial	103.6	2.15	2	9.830	-
475506756	glutaredoxin-3	36.6	10.67	3	10.845	-
971406204	PREDICTED: glycerol-3-phosphate dehydrogenase 1-like protein	38.4	14.90	4	10.938	-
82106351	RecName: Full=Protein deglycase DJ-1	19.9	11.64	2	11.067	-
539389972	glyoxalase domain-containing protein 4	34.5	7.05	2	12.234	-
308818127	3'(2'),5'-bisphosphate nucleotidase 1	32.9	10.10	3	12.249	-
971443636	PREDICTED: 4-trimethylaminobutyraldehyde dehydrogenase-like	38.6	8.81	2	12.527	-
227016	apolipoprotein AI	28.8	23.17	5	12.710	-
399491	RecName: Full=Fibrinogen beta chain; Contains	52.6	6.05	2	12.836	-
480354971	acetyl-CoA acetyltransferase, mitochondrial	44.1	14.73	4	13.567	-
971436124	PREDICTED: endophilin-A2 isoform X3	40.7	8.94	3	15.331	-
71896205	isocitrate dehydrogenase [NADP], mitochondrial	50.4	11.50	4	15.746	-
118104602	PREDICTED: alpha-aminoadipic semialdehyde dehydrogenase	58.0	7.09	3	16.313	2
123907663	RecName: Full=1-phosphatidylinositol 4,5-bisphosphate phosphodiesterase zeta-1	72.5	3.30	2	17.350	-
303227895	putative transferase CAF17, mitochondrial	36.1	7.83	2	17.664	-
1536812	immunoglobulin heavy chain variable region, partial	10.8	28.85	2	18.660	-
122692295	ubiquitin carboxyl-terminal hydrolase isozyme L1	25.1	12.95	2	18.800	-
288562779	Chain A, Hen Egg White Lysozyme E35q Chitopentaose Complex	14.3	21.71	2	18.852	-
118082453	PREDICTED: acyl-CoA synthetase short-chain family member 3, mitochondrial	73.4	2.84	2	19.043	-
971376395	PREDICTED: pseudouridine-5'-phosphatase	26.2	7.66	2	19.070	-

614458442	immunoglobulin Y heavy chain constant region, partial	42.6	7.73	2	19.147	-
310772215	phosphatidylethanolamine-binding protein 1	20.9	22.99	3	19.225	-
971399007	PREDICTED: creatine kinase B-type isoform X1	42.2	14.36	5	19.986	-
57525373	leukotriene A-4 hydrolase	69.3	4.41	2	20.623	-
971384682	PREDICTED: ribonuclease UK114	14.8	15.11	2	20.668	-
1334630	unnamed protein product	28.0	8.73	2	20.679	-
89000599	immunoglobulin light chain variable region, partial	12.2	22.69	2	20.815	-
45383738	aconitate hydratase, mitochondrial	85.7	6.24	4	21.215	-
971373618	PREDICTED: alpha-2-macroglobulin-like protein 1 isoform X4	145.7	2.63	3	21.754	-
54036927	RecName: Full=Cytochrome c	11.7	15.24	2	22.260	-
971370356	PREDICTED: dihydrolipoyl dehydrogenase, mitochondrial isoform X1	53.9	11.22	4	22.278	-
71897293	2-oxoglutarate dehydrogenase, mitochondrial	115.1	2.76	2	23.870	-
50761844	PREDICTED: tubulin polymerization-promoting protein family member 2	21.5	28.36	5	23.894	-
971448230	PREDICTED: vitelline membrane outer layer protein 1 homolog	21.0	17.68	3	24.045	-
50758110	PREDICTED: malate dehydrogenase, mitochondrial	36.9	29.63	10	24.848	-
1708509	RecName: Full=Ovoinhibitor; Flags: Precursor	51.9	9.32	4	25.010	1
52695547	Chain B, Understanding Protein Lids: Structural Analysis Of Active Hinge Mutants In Triosephosphate Isomerase	26.5	20.65	4	25.185	-
45384204	10 kDa heat shock protein, mitochondrial	11.1	21.57	2	26.262	-
153792243	polymeric immunoglobulin receptor precursor	70.5	5.09	3	26.310	2
971378941	PREDICTED: hemopexin	49.6	5.78	2	29.334	1
1706653	RecName: Full=Alpha-enolase; AltName: Full=2-phospho-D-glycerate hydro-lyase	47.3	19.35	7	29.541	-
57529797	long-chain specific acyl-CoA dehydrogenase, mitochondrial	47.9	10.90	3	29.802	-
971443790	PREDICTED: guanosine-3',5'-bis(diphosphate) 3'-pyrophosphohydrolase MESH1-like	20.6	11.35	2	30.789	-

971434946	PREDICTED: EF-hand calcium-binding domain-containing protein 3 isoform X3	97.4	1.82	2	30.816	-
37926870	Chain A, Diferric Chicken Serum Transferrin At 2.8 A Resolution.	75.8	15.16	10	32.751	-
971451669	PREDICTED: electron transfer flavoprotein subunit beta, partial	21.4	12.44	2	33.055	1
766944282	serum albumin precursor	69.8	32.20	19	33.494	-
57524920	glucose-6-phosphate isomerase	62.2	8.68	4	33.681	-
475807703	enoyl-CoA hydratase, mitochondrial precursor	31.6	6.64	2	34.655	-
971413980	PREDICTED: aspartate aminotransferase, mitochondrial isoform X1 [	38.8	13.83	4	35.390	-
478431053	enoyl-CoA delta isomerase 1, mitochondrial	34.3	14.61	4	35.599	-
363734612	PREDICTED: plasma protease C1 inhibitor	54.4	4.97	2	36.265	1
57530355	malate dehydrogenase, cytoplasmic isoform MDH1	36.5	15.27	5	37.173	-
57530433	fumarate hydratase, mitochondrial	54.3	7.89	3	37.527	-
971374570	PREDICTED: omega-amidase NIT2 isoform X1	31.0	13.48	4	37.753	-
6920068	RecName: Full=L-lactate dehydrogenase B chain;	36.3	28.23	7	37.984	-
46395491	PIT54 protein precursor	50.8	12.13	4	38.259	-
971419880	PREDICTED: 4-aminobutyrate aminotransferase, mitochondrial	56.5	7.00	3	38.839	-
513168207	PREDICTED: peroxisomal carnitine O-octanoyltransferase	70.0	8.50	4	41.691	-
223208	aminotransferase,Asp	45.5	11.71	4	46.996	-
82233833	RecName: Full=Isoleucine--tRNA ligase, mitochondrial	111.9	3.70	3	58.530	-
86129492	pantetheinase precursor	55.4	8.67	4	64.483	1
971451037	PREDICTED: lysosomal alpha-mannosidase-like, partial	45.5	4.78	2	64.530	-
126046	RecName: Full=L-lactate dehydrogenase A chain	36.5	12.95	3	67.239	-
971373694	PREDICTED: ovostatin	169.4	1.47	2	71.388	-
971450173	PREDICTED: fructose-bisphosphate aldolase A-like, partial	21.2	22.56	2	71.972	-
971370928	PREDICTED: protein NEL isoform X1	85.9	8.15	5	72.538	1
45383183	dihydropyrimidinase-related protein 3	62.0	4.74	2	72.538	-

D. Non-raft specific proteins

Accession	Description	MW	Coverage	Pep[1]	Ratio[2]	TM[3]
487441312	prefoldin subunit 4	15.4	20.15	2	-	-
82194894	RecName: Full=Ubiquitin-conjugating enzyme E2 variant 2	16.3	19.44	2	-	-
971398440	PREDICTED: synaptosomal-associated protein 23 isoform X1	23.6	12.92	2	-	-
62972	proteasome C1 subunit, partial	21.8	12.75	2	-	-
57525182	26S proteasome non-ATPase regulatory subunit 9	22.7	11.59	2	-	-
71897167	proteasome subunit alpha type-5	26.4	10.79	2	-	-
82074965	Cell cycle control protein 50A	41.4	9.68	2	-	2
971427860	PREDICTED: guanine nucleotide-binding protein G(s) subunit alpha	35.9	9.51	2	-	-
73919467	RecName: Full=Surfeit locus protein 1	35.2	9.39	2	-	-
71897053	flotillin-2	36.2	9.39	3	-	-
971374423	PREDICTED: leukocyte surface antigen CD47 isoform X3	34.8	9.18	2	-	6
971436173	PREDICTED: small glutamine-rich tetratricopeptide repeat-containing protein alpha isoform X2	33.8	8.97	2	-	-
971445900	PREDICTED: delta(3,5)-Delta(2,4)-dienoyl-CoA isomerase, mitochondrial	33.6	8.12	2	-	-
295148230	prohibitin	29.9	6.99	2	-	-
2392240	Chain D, Mitochondrial Creatine Kinase	43.2	6.84	2	-	-
971443719	PREDICTED: probable phospholipid-transporting ATPase IF	35.1	6.82	2	-	2
971438308	PREDICTED: NADH dehydrogenase [ubiquinone] iron-sulfur protein 4, mitochondrial isoform X1	32.7	6.80	2	-	-
906848896	Chain N, Cytochrome Bc1 Complex From Chicken With Designed Inhibitor Bound	49.4	6.50	3	-	-
971381407	PREDICTED: metalloreductase STEAP4	53.4	5.51	3	-	6
513188218	PREDICTED: methylmalonate-semialdehyde dehydrogenase [acylating], mitochondrial	58.3	5.20	2	-	-

971437898	PREDICTED: aquaporin-7 isoform X5	30.3	4.95	2	-	3
971405727	PREDICTED: calcium-binding mitochondrial carrier protein Aralar1 isoform X5	70.7	4.36	2	-	-
971392324	PREDICTED: long-chain-fatty-acid--CoA ligase 4 isoform X2	74.5	4.18	2	-	-
971408542	PREDICTED: nexilin isoform X9	72.3	4.09	2	-	-
971410136	PREDICTED: long-chain-fatty-acid--CoA ligase 3	79.3	3.93	2	-	1
971440289	PREDICTED: methylcrotonoyl-CoA carboxylase beta chain, mitochondrial	60.6	3.93	2	-	-
497241981	beta-galactosidase preproprotein	73.4	3.53	2	-	-
45382957	prothrombin precursor	69.1	3.29	2	-	-
513213432	PREDICTED: (E3-independent) E2 ubiquitin-conjugating enzyme isoform X2	131.2	3.10	2	-	-
971376924	PREDICTED: probable phospholipid-transporting ATPase IH isoform X8	130.1	1.76	2	-	8
971440604	PREDICTED: adenylate cyclase type 10-like	160.0	1.57	2	-	1
971434955	PREDICTED: alpha-2-macroglobulin-like protein 1	163.2	1.49	2	-	1
513165569	PREDICTED: complement C4	189.9	1.24	2	-	-
971389662	PREDICTED: collagen alpha-1(XII) chain isoform X2	333.3	0.82	2	-	-
513208830	PREDICTED: tektin-5	55.6	5.59	2	-	-
57529492	3-ketoacyl-CoA thiolase, mitochondrial	41.7	5.54	2	-	-
971421650	PREDICTED: tectonic-2 isoform X2	74.6	5.51	2	-	2

[1]Number of unique peptides

[2] Relative protein quantity of Non-raft to DRM

[3]Number of transmembrane domains
